# Rewiring the brain: the AI revolution in epilepsy treatment

**DOI:** 10.3389/fneur.2026.1781782

**Published:** 2026-05-07

**Authors:** Kismatdeep Kahlon, Arany Umasuthan, Nilakshi Debnath

**Affiliations:** University of Limerick, Limerick, Ireland

**Keywords:** artificial intelligence, convolutional neural network (CNN), EEG, epilepsy, machine learning (ML), surgical localization, wearable devices, pediatric epilepsy

## Introduction

Epilepsy is a prevalent neurological disorder, with an estimated five million new diagnoses annually (1). With ongoing advancements in technology, Artificial Intelligence (AI) has been explored for its potential applications in personalized epilepsy treatment, guiding surgical decisions, and enhancing neurostimulation. AI involves using computer systems and machines to perform tasks that typically require human intelligence. Machine Learning (ML) is a subset of AI in which algorithms learn from data analysis, identifying patterns and generating predictive outcomes.

Contributions from AI algorithms can assist with treatment options for drug-resistant epilepsy (DRE), defined as persistent seizures despite two adequate anti-seizure medication trials. AI can additionally identify precise epileptogenic zones, and analyze electroencephalograms (EEGs), allowing for optimal management and prevention. Yet much of the current literature presents AI in epilepsy as a broad field of promise rather than clarifying what is ready for clinical integration and what remains experimental.

In this opinion article, we argue that AI in epilepsy demonstrates variable levels of clinical readiness across domains of care. AI-assisted EEG analysis and seizure detection are approaching clinical readiness as supervised decision-support tools, whereas AI-driven treatment selection, surgical outcome prediction, and adaptive neuromodulation remain investigational. Safe adoption will require prospective validation and regulatory oversight. Rather than replacing clinician expertise, AI should be cautiously integrated as an adjunct within structured clinical governance frameworks.

## AI-assisted EEG interpretation

Among all proposed applications, AI-assisted EEG interpretation has the strongest case for near-term clinical integration as a supervised decision-support tool. Continuous EEG monitoring produces volumes of data that exceed the capacity of manual review alone, creating a clear operational need for algorithmic triage and pattern recognition.

High performance has been demonstrated in benchmark datasets. For example, a deep learning (DL) model evaluated on the Bonn and New Delhi EEG datasets achieved classification accuracies of 99.9%−100%, with reported sensitivity, specificity, and precision approaching 100% in interictal–ictal discrimination tasks (2). The authors emphasized the model's computational speed and potential to reduce human screening burden in large EEG datasets; however, these results were obtained in controlled datasets rather than diverse clinical populations, and therefore broader validation remains necessary (2).

More clinically representative evidence comes from large multicentre validation efforts. In a diagnostic accuracy study, a convolutional neural network (CNN) model (SCORE-AI) was developed using 30,493 routine EEG and validated across three independent datasets (3). In a representative multicentre test set, SCORE-AI achieved a specificity of 90%, significantly higher than the consensus of three human experts (73.3%), while maintaining comparable sensitivity (86.7% vs. 93.3%) and overall accuracy (88.3% vs. 83.3%) (3). These findings demonstrate that, in defined populations, fully automated EEG interpretation can achieve human-expert–level performance, particularly improving specificity and interpretive consistency.

In the intensive care unit (ICU) EEGs, SPaRCNet classified ICU EEG patterns (including seizures and seizure-like events) using over 6,000 EEGs from 2,700 patients, and were compared against 20 fellowship-trained neurophysiologists (4). The model matched or exceeded most experts in sensitivity, specificity, and calibration across multiple clinically relevant patterns (4). This demonstrates AI can handle complex EEG tasks with expert-level performance when trained on large, carefully annotated datasets.

In pediatrics, neonatal seizures often occur without the overt physical manifestations seen in older children and adults, requiring expert EEG monitoring for accurate detection, though such expertise may not always be readily available (5). Recent work in neonatal seizure detection demonstrates how large-scale datasets can substantially improve algorithmic reliability. A CNN trained on over 12,000 seizure events achieved state-of-the-art performance, evaluated using balanced metrics for both continuous and binary variables with expert-level agreement across independent validation datasets (6). Notably, inter-rater agreement did not significantly differ when the model replaced a human reviewer.

Taken together, these findings suggest that AI-assisted EEG analysis is approaching clinical readiness not as autonomous systems, but rather as a triage and decision-support under neurologist supervision. The strongest current use case lies in improving efficiency, reducing review time, and enhancing interpretive consistency in routine EEG workflows.

## Surgical localizations

AI applications in epilepsy surgery, including seizure onset zone localization and prediction of postoperative seizure freedom, are highly developed and approaching clinical applicability, though they remain investigational. Anterior temporal lobectomy (ATL) is an effective option for select patients, but surgical selection can be challenging, and the procedure carries inherent risks and costs. Early studies have explored whether ML can improve patient selection and outcome prediction.

In one study, a simulated neural network (SNN) was trained on data from 87 patients across three surgical centers to predict post-ATL seizure outcomes (7). When compared with conventional discriminant function analysis, the SNN achieved higher predictive accuracy of 81.8% for seizure-free outcomes and 95.4% for nearly or totally seizure-free outcomes (7). These results suggest that AI could support more precise, individualized surgical planning.

Similarly, ML models using clinical and neuropsychological features were able to predict seizure recovery in patients with temporal lobe epilepsy secondary to hippocampal sclerosis with nearly 90% estimated accuracy (8). Some features, such as personality style, a variable which quantifies cognitive-perceptive responses of an individual, emerged as particularly important, while others were irrelevant to prognosis. Although these analyses were conducted in relatively small cohorts, the findings were internally validated (8). External validation on larger, multicentre cohorts remains a key next step for complete clinical readiness.

Neuroimaging-based ML models are publicly available for automated lesion detection with magnetic-resonance imaging (MRI), particularly useful in focal cortical dysplasia (FCD) which is a common cause of DRE among children and difficult to localize (9). Kersting et al., evaluated current models including, Morphometric Analysis Program 2018 (MAP18), Multi-centre Epilepsy Detection (MELD), DL-based model for FCD detection (deepFCD) with multicentre datasets and developed new models, supporting improved lesion localization for surgical planning.

## Implantable and wearable devices

In contrast to surgical AI, ML-driven implantable neurostimulation devices remain early-stage and highly investigational. A closed-loop system includes a signal acquisition interface, a processing unit for feature extraction, and a stimulation module for electrical feedback (10). These systems aim to detect early seizure signatures and deliver targeted stimulation in real time.

One such example is NET-TEN, a neuromorphic processor designed to detect seizure-like activity directly from brain signals (10). By mimicking biological neurons, NET-TEN can identify pathological activity on-chip, and filter out artifacts, demonstrating proof-of-concept that AI can enable rapid, data-driven detection at the edge (10). These features could eventually support closed-loop seizure intervention, where abnormal activity is detected and disrupted automatically. However, in its current state, the device does not yet reliably differentiate ictal (seizure) from interictal events and remains susceptible to electrical artifacts; nonetheless, it represents a promising proof of concept for real-time neuromorphic seizure detection with potential future clinical applications.

In parallel, wearable monitoring technologies are being developed to improve seizure detection and documentation. By embedding ML algorithms onto neuromorphic chips, researchers have shown the feasibility of real-time, patient-specific seizure forecasting that could eventually alert individuals to impending seizures and generate rich longitudinal data on seizure patterns for clinicians (11). The commercially available NightWatch detection bracelet has demonstrated a median sensitivity of 100% for detecting nocturnal major motor seizures in children aged 4–16 when evaluated alongside video recordings, but remains investigational (12).

Together, implantable and wearable systems represent complementary approaches: implantable devices aim to intervene in real time, whereas wearables primarily focus on detection and monitoring, both supported by advances in AI.

## Discussion

AI is increasingly being integrated into epilepsy research and clinical workflows, yet its readiness for clinical adoption varies substantially across applications. Our opinion suggests that AI-assisted EEG interpretation and seizure detection currently represent the most clinically mature applications, while additional AI algorithms remain investigational.

Despite promising performance in controlled research settings, several barriers continue to limit the broader clinical adoption of AI in epilepsy care. A significant challenge is the limited generalizability of many AI algorithms. Diagnostic models typically require large datasets and extensive patient-specific data, yet, in practice, variability exists across equipment, EEGs, and imaging, and patient demographics as well (13). These factors reduce the real-world applicability of many AI algorithms as models trained on specific populations often perform poorly when applied across different clinical environments. Although ML algorithms may support earlier diagnosis and precise treatment planning where specialist expertise is scarce, a study done by Duta et al. (14) found constricted generalizability of clinical epilepsy diagnostic tools among low to middle income countries, utilizing models which were developed from different populations. This is of concern as many ML models have been developed using data from a single region and inadequate representation of certain demographic groups may introduce algorithmic bias, potentially resulting in exacerbation of existing healthcare disparities (13). This warrants caution of unequal performance across populations, as well as unpredictability and misdiagnoses, highlighting the need for diverse, geographically representative training datasets to ensure equitable performance across global populations.

To ensure generalizability, there is a necessity of external validation using multicenter datasets prior to widespread implementation. An external validation study found that the SCORE-AI model has demonstrated generalizability across geographically distinct populations when evaluated with externally collected data and frozen model parameters (15). However, many existing AI algorithms are developed using relatively small, adult-focused datasets, which may limit their reliability in pediatric populations. Although SCORE-AI was designed for individuals older than 3 months, the external validation cohort primarily consisted of participants over the age of 15, highlighting the need for further evaluation in younger age groups. Expanding pediatric representation in training and validation datasets is therefore essential, particularly for vulnerable populations and individuals with comorbidities. For example, individuals with intellectual disability and epilepsy can experience more severe and treatment-resistant seizures, yet long-term datasets for this group remain limited (16). Communication barriers and higher rates of misdiagnosis further complicate their clinical management. Ethical strategies, such as employing adapted communication methods for consent during data collection, have been proposed to improve inclusion in AI research (16). Increasing the representation of these populations in model development and validation is critical to reduce bias and improve diagnostic accuracy in AI-assisted epilepsy care.

In addition to ensuring inclusion of pediatric populations in validation studies, attention must be paid to consequences of inaccurate detection. Although some commercially available wearable devices have high sensitivity rates, there are concerns of false positive notifications. The wearable NightWatch assisted with detection of nocturnal seizures, however, false alarm rates were still present, and particularly in children with learning disabilities for seizures that were detected as not clinically urgent (12). Misdiagnoses and false alarms can induce anxiety and social isolation, especially in pediatric populations (17). It is essential to utilize pediatric datasets to mitigate potential for biases as well as reduce inappropriate diagnoses and treatment options (17).

Ethical considerations also play an important role in the responsible integration of AI in epilepsy. Additional consideration of data privacy is important as most data in AI models contain sensitive patient information, including seizure histories, EEG recordings, and treatment responses. Ensuring compliance with guidelines and regulations like the General Data Protection Regulation (GDPR) in Europe and the Health Insurance Portability and Accountability Act (HIPAA) in the United States is critical for ethical and transparent clinical integration (13). The risk of data breaches and unauthorized access increases as the volume of collected data increases, requiring tougher security safeguards. There are additional considerations of autonomy as AI systems are inherently complex, and it is important to establish transparent decision-making with patients and caregivers to establish trust and incorporate effective clinical oversight (17). Transparency is critical in algorithm design and utilization to maintain trust among clinicians and patients, and should be multilayered with developers, clinicians, institutions, and regulators (18). Although access to larger data sets will help assess the reliability of ML algorithms, this may affect patients' rights for confidentiality and require extensive transparency for informing patients of how their data will be stored and utilized with AI (19).

Successful clinical integration of AI requires rigorous validation, interdisciplinary collaboration, equitable data representation, and careful ethical oversight, particularly in pediatric populations. While AI has the potential to reduce cognitive burden, there are additional risks of over-reliance which may consequently diminish clinical judgment over time (17). Final therapeutic decisions must remain grounded in clinical expertise, and thoughtful integration of AI technologies may significantly enhance future epilepsy care.
